# Robenacoxib versus meloxicam for the management of pain and inflammation associated with soft tissue surgery in dogs: a randomized, non-inferiority clinical trial

**DOI:** 10.1186/1746-6148-9-92

**Published:** 2013-05-02

**Authors:** Philippe Gruet, Wolfgang Seewald, Jonathan N King

**Affiliations:** 1 Novartis Animal Health Inc., Lead Finding, Basel, CH-4058, Switzerland; 2 Novartis Animal Health Inc., Clinical Development, Basel, CH-4058, Switzerland

**Keywords:** Analgesia, Dog, Meloxicam, Peri-operative, Robenacoxib, Soft tissue surgery

## Abstract

**Background:**

Non-steroidal anti-inflammatory drugs (NSAIDs) are used routinely to control pain and inflammation after surgery in dogs. Robenacoxib is a new NSAID with high selectivity for the cyclo-oxygenase (COX)-2 isoform of COX. The objective of this study was to evaluate the efficacy and tolerability of robenacoxib for the management of peri-operative pain and inflammation associated with soft tissue surgery in dogs. The study was a prospective, randomized, blinded, positive-controlled, non-inferiority, multi-center clinical trial. A total of 174 dogs undergoing major soft tissue surgery were included and randomly allocated in a 2:1 ratio to receive either robenacoxib (n = 118) or the positive control, meloxicam (n = 56). Each dog received an initial dose subcutaneously prior to surgery (robenacoxib 2 mg/kg, meloxicam 0.2 mg/kg), followed by daily oral doses (robenacoxib 1–2 mg/kg, meloxicam 0.1 mg/kg) for 12 days (range 10–14) after surgery. Pain and inflammation were assessed subjectively using the Glasgow Composite Pain Scale (GCPS) by clinicians as the primary end point and additional evaluations by the clinicians and animal owners as secondary endpoints.

**Results:**

Both treatments provided similar pain control, with no significant differences between groups for any efficacy variable using non-parametric analyses (Mann–Whitney *U* test). In no dog was analgesic rescue therapy administered. Non-inferior efficacy of robenacoxib compared to meloxicam was demonstrated statistically for the primary and all secondary endpoints using parametric analysis of variance, although the data were not normally distributed even after log transformation. For the primary endpoint (reciprocal of the modified GCPS score), the relative efficacy of robenacoxib/meloxicam was 1.12 with a 95% confidence interval of 0.97-1.29.

Both treatments were well tolerated and did not affect buccal mucosal bleeding time.

**Conclusion:**

A treatment regimen of robenacoxib by subcutaneous injection followed by oral tablets had good tolerability and non-inferior efficacy compared to meloxicam for the management of peri-operative pain and inflammation associated with soft tissue surgery in dogs.

## Background

Soft tissue surgical procedures in the dog are associated with post-operative pain and peri-operative analgesia is therefore recommended in many cases [1,2]. The duration of pain control required varies between cases, but in some instances is needed for seven days or longer [1]. The most frequently used analgesics in dogs are opioids and non-steroidal anti-inflammatory drugs (NSAIDs) [2]. The introduction of newer NSAIDs which are selective inhibitors of the cyclooxygenase (COX)-2 isoform of COX (COX-2) should permit relief of pain and inflammation whilst minimizing the adverse effects of non-selective COX inhibitors in causing gastrointestinal tract damage and interfering with blood clotting pathways [3].

Robenacoxib is a new NSAID with several properties of interest for use in dogs undergoing surgery, including a fast onset of action and the availability of both injection and oral formulations [4]. Robenacoxib has a high safety index in healthy dogs, which is attributed to its pharmacodynamic and pharmacokinetic properties [5]. First, robenacoxib is highly selective for COX-2 in dogs, and at recommended dosages inhibits COX-2 while sparing COX-1 [4,6]. Second, robenacoxib is cleared rapidly from the central body compartment, but persists at sites of inflammation [3,7]. The efficacy and tolerability of robenacoxib has been demonstrated in dogs undergoing orthopedic surgery [8].

In this study, the efficacy and tolerability of robenacoxib were evaluated in dogs undergoing soft tissue surgery. Since other NSAIDs are already registered in the European Union (EU) for this indication, and widely used, the study was a non-inferiority comparison to a positive control, meloxicam. Meloxicam was selected as it is registered in the EU for the reduction of post-operative pain and inflammation following soft tissue (and orthopedic) surgery after a single injection pre-surgery, used extensively, and its efficacy is proven for soft tissue surgery pain in dogs [9-11]. The hypothesis of the study was that robenacoxib would have non-inferior efficacy and tolerability compared to meloxicam.

## Methods

### Experimental design

The study was a prospective, randomized, parallel-group, blinded, multi-center clinical trial conducted at 16 French and 9 German veterinary practices. The study schedule is shown in Table 1. The study was approved by the French and German regulatory authorities and Novartis committees taking into account scientific, ethical and animal welfare guidelines. The investigation was conducted in compliance with Good Clinical Practice (VICH GL9, CVMP:VICH/595/98, 2000).

**Table 1:** Study schedule

**Time (T)**	**T0**	**T1**	**T1 + 1 h**	**T1 + 2 h**	**T1 + 4 h**	**T1 + 8 h**	T1 + 24 h* (Day 1)	Day 12*
Examination number/event	1 (initial visit)	Extubation	2	3	4	5	6	7
Drug treatment	Subcutaneous dose **	-	-	-	-	-	First oral dose	Final oral dose
Blood sample number for clinical chemistry and hematology	1	-	-	-	-	-	2	3

* Each dog was dosed orally once daily up to Day 12 (range 10–14) when the stitches were removed.

** Administered prior to surgery, close to the time of induction of anesthesia.

### Animals

All owners gave written informed consent for their dogs to be included in the study. The inclusion criteria comprised dogs aged ≥ 6 weeks, either gender, any breed, weighing between 2.5 and 80 kg, scheduled to undergo major soft tissue surgery. Exclusion criteria comprised dogs: known to be pregnant or lactating; with severe concomitant disorders (gastro-intestinal tract, kidney or liver) that may have interfered with the evaluation of response to treatment; which received local or systemic NSAIDs or opioids within 24 hours, or corticosteroids within 30 days, prior to inclusion in the study. Exclusion criteria after inclusion and first dosing were: concomitant disorders that could interfere with evaluation of response to treatment (e.g. trauma); forbidden concomitant treatment; adverse events requiring cessation of treatment.

### Randomization and treatment allocation

Dogs were allocated randomly to the two treatment groups in a 2:1 ratio using computer generated randomization lists. The 2:1 ratio was used to obtain data in more dogs receiving robenacoxib and was predicted to cause only a modest (approximately 10%) reduction in statistical efficiency compared to a 1:1 ratio. Case allocation was stratified according to investigator and anticipated duration of surgery at two levels, < 1 hour and > 1 hour.

### Test articles and blinding

Dogs in group 1 received robenacoxib (Onsior® 20 mg/mL Solution for Injection, Onsior® 5 or 20 mg Tablets, Novartis Santé Animale, Huningue, France) at a dosage of 2 mg/kg subcutaneously as an initial dose on day 0, and then 1–2 mg/kg orally once daily starting on day 1 for 12 days (range 10–14 days). The treatment time was selected since previous work indicated that pain can persist for 10–14 days after soft tissue surgery in dogs [1]. The tablets could be administered with or without food, with no specific instructions regarding feeding. Co-administration of Onsior® tablets with the entire daily ration in dogs leads to a slight (26%) reduction in bioavailability compared to administration without food [12].

Group 2 dogs were administered meloxicam (Metacam® 5 mg/mL solution for injection, Labiana Life Sciences S.A., Terrassa, Spain and Metacam® oral suspension, Boehringer Ingelheim GmbH, Ingelheim/Rhein, Germany) at a dosage of 0.2 mg/kg by subcutaneous injection on day 0, and then 0.1 mg/kg orally mixed with food once daily starting on day 1 for up to 12 days (range 10–14 days).

As robenacoxib and meloxicam formulations differed, blinding was maintained by the “double-investigator technique”: one investigator, the clinician, was responsible for clinical assessments and another, the dispenser, was responsible for treatment administration, prescription and compliance control. Owners were not blinded to treatment group.

### Concomitant treatments

Drugs which could affect efficacy assessments were disallowed, including: analgesics (opioids and α_2_-agonists), other NSAIDs, corticosteroids, macrolides and tetracyclines. Opioids were not used as premedication as this was not routine practice in France and Germany at the time the study was conducted. However rescue analgesic therapy (of any type) was permitted at any time if the clinician judged it was necessary. Since use of analgesic therapy was allowed for animal welfare reasons, and was not a specific predefined efficacy endpoint, no specific criteria were defined in the protocol when rescue therapy should be used. Standard parameters relating to anesthetic premedication, induction, maintenance and recovery were recorded.

### Efficacy assessment

Investigators were instructed that the same clinician (a veterinarian) should make all efficacy assessments for all cases at each site, whenever possible. The frequency of exemptions to this rule was not assessed. The clinicians were trained on the efficacy scoring methods before the start of the study with the objective to standardize assessments. However no validation, for example no assessment of intra and inter-observer variation, was made.

The primary endpoint for efficacy comprised the Glasgow Composite Pain Scale (GCPS) [13], assessed by the clinician at seven examination times (T, hours) as indicated in Tables 1 and 2: T1 (extubation), T1 + 1 h, T1 + 2 h, T1 + 4 h, T1 + 8 h, T1 + 24 h and day (D)12. For the first 6 examinations all components (A1, A2, B, C, D1 and D2) of the scale were recorded. For the final examination at D12, parts B, C, D1 and D2 were recorded. Part B (mobility) could not be assessed in 21 dogs at early time points. As it is not valid to compare dogs for which data with mobility was scored or not scored, the main primary endpoint for the study was the “modified” GCPS score without part B (n = 153 dogs, maximum score 20).

**Table 2:** The Glasgow Composite Pain Scale (GCPS)* used by the clinicians to assess the dogs

**Part**	**Circumstance**	**Assessment and Scale**
A1	Dog in kennel	Vocalization: Is the dog:
		[0] quiet
		[1] crying or whimpering
		[2] groaning
		[3] screaming
A2		Attention to wound area: Is the dog:
		[0] ignoring any wound or painful area
		[1] looking at wound or painful area
		[2] licking wound or painful area
		[3] rubbing wound or painful area
		[4] chewing wound or painful area
B	Dog out of kennel on lead	Mobility: When the dog rises/walks is it:
		[0] normal
		[1] lame
		[2] slow or reluctant
		[3] stiff
		[4] it refuses to move
C	Response to touch	Response to touch: does the dog:
		[0] do nothing
		[1] look around
		[2] flinch
		[3] growl or guard area
		[4] snap
		[5] cry
D1	Overall assessment	Demeanor: Is the dog:
		[0] happy and content or happy and bouncy
		[1] quiet
		[2] indifferent or non-responsive to surroundings
		[3] nervous or anxious or fearful
		[4] depressed or non-responsive to stimulation
D2		Posture: Is the dog:
		[0] comfortable
		[1] unsettled
		[2] restless
		[3] hunched or tense
		[4] rigid

* Parts A, B, C and D were performed sequentially at the first 6 examination times listed in Table 1. For examination 7 on Day 12, only parts B, C and D were used.

There were six secondary efficacy endpoints, as follows:

(1 and 2) Using Visual Analogue Scales (VAS) at seven examination times (Table 1) the clinician assessed: (a) pain at rest on a scale ranging from 0 (no pain) to 100 (severe pain manifested by vocalization, aggression, refusal to allow examination); and (b) pain during gentle palpation/manipulation of the affected limb/joint on a scale ranging from 0 (no pain elicited) to 100 (severe pain manifested by vocalization, aggression, refusal to allow examination) [14].

(3) Clinician’s global assessment of efficacy (overall pain control) at T1 + 24 h on the scale 0 = excellent, 1 = good, 2 = fair, 3 = poor.

(4) Clinician’s assessment of inflammation, using a VAS, and based on joint/limb swelling, local heat, redness or paresis, ranging from 0 (no inflammation) to 100 (major inflammation) on D12.

(5 and 6) Owner’s assessment of the dog daily from D1 to D12 using scales for: (a) demeanor and (b) mobility. The scores were: 0 = normal and 1–4 for slight (1), moderate (2), marked (3) or severely (4) modified demeanor or impaired mobility. Mobility could not be assessed in all dogs.

### Plasma cortisol concentration

Venous blood samples (1 mL) were collected into tubes containing EDTA for measurement of plasma cortisol concentrations at the times T0 (before surgery), T1 (extubation), T1 + 1 h, T1 + 2 h, T1 + 4 h and T1 + 8 h (Table 1). Plasma samples were stored at -20°C prior to analysis. Cortisol was measured by radio-immunoassay using a commercial kit (IM-1841, Immunotech, Marseille, France) at the National Veterinary School of Toulouse, France. Within-day and day-to-day precisions were less than 14% and the accuracy was within the range 93-109%. The limit of quantification of the assay was 10 ng/mL.

### Tolerability assessment

The tolerability of administered treatments was assessed from reported adverse events, clinical examination, pain at injection, clinical chemistry, hematology and buccal mucosal bleeding time. The clinicians and dog owners were informed that two NSAIDs were being tested, and that adverse effects of NSAIDs affect most frequently the gastrointestinal tract, kidney and liver. No specific assessment was made of sedation. Pain at the time of injection (T0) was assessed by the dispenser on a scale ranging from 0 (no pain) to 3 (severe pain).

Venous blood samples (5 mL) were collected at three times (T0, T1 + 24 h and D12, Table 1) for measurement of variables including serum activities of alanine aminotransferase, alkaline phosphatase, aspartate aminotransferase, gamma glutamyltransferase and concentrations of creatinine, total protein and urea. Venous blood samples (2 mL) were collected into EDTA at the same times for measurement of hematology variables including red blood cell, white blood cell and differential white blood cell counts, hematocrit and hemoglobin concentration. Buccal mucosal bleeding times were measured at times T0 and T1 + 24 h.

### Statistical analyses

Statistical tests were performed using SAS® Software, Version 8.2 (Cary, NC, US). All tests were performed two-sided on a 5% level of significance. The main analyses were conducted on the intention-to-treat population (i.e. all randomized animals). The study was planned for a minimum of 150 dogs (100 receiving robenacoxib and 50 receiving meloxicam). The sample size was calculated for 80% power in the non-inferiority analysis of the primary endpoint (GCPS scores) using data from a pilot study. For summary statistics, non-parametric and analysis of variance (ANOVA) statistical tests, missing values were imputed using the last observation carried forward method (LOCF). The LOCF method was not used for repeated measures ANOVA (RMANOVA), which was used to analyze most efficacy endpoints. For the primary endpoint, the GCPS scores, data were missing from a total of 4 dogs (2 with robenacoxib, 2 with meloxicam).

#### Initial comparability tests

Demographic and baseline data were compared between groups using the Mann–Whitney *U* test for ordinal (e.g. body weight, effective duration of surgery) or binary data (e.g. sex), and with the Kruskal-Wallis test for non-binary nominal data (e.g. combination of sex and neutered status).

#### Efficacy variables

Efficacy measures and plasma cortisol concentrations were analyzed statistically after log transformation using ANOVA for variables with a single time point post-treatment or RMANOVA if there were multiple time points, as for most variables. The initial RMANOVA model included the following variables: treatment group, time, time/treatment interaction, baseline value (where applicable), foreseen duration of the surgery, duration of intubation, time between administration of test treatment and extubation, sex, neutered status, body weight, age, wakening time, country and type of surgery. Variables (except treatment group) were removed progressively from the model if *P* > 0.3.

Non-inferiority was defined if the 95% confidence interval of the efficacy of robenacoxib divided by that of meloxicam was greater than the pre-defined threshold of 1-σ, with σ = 0.20. Reciprocal pain scores were used, so that the lower limit of the 95% confidence interval for (1/mean score robenacoxib)/(1/mean score meloxicam) needed to completely lie above 0.80.

Since most efficacy variables were not normally distributed even after log transformation (*P* < 0.05 using the Shapiro-Wilk test), groups were also compared using the non-parametric Mann–Whitney *U* test in addition to the ANOVA/RMANOVA analyses.

#### Clinical chemistry, hematology and bleeding time

As normality of these data was not proven, non-parametric methods were applied. The Mann–Whitney *U* test was used to compare the two groups before surgery, after surgery, and the change between before and after surgery. In addition, data before surgery and after surgery were compared using the Wilcoxon paired-samples test, separately for each group.

### Adverse events

The incidence of adverse events in the two groups was compared with the Fisher Exact test.

## Results

### Animals

A total of 174 client owned dogs were recruited from clinical cases scheduled to undergo major soft tissue surgery. Unless stated all reported results refer to the “intention-to-treat” population, which consisted of all 174 dogs which were randomized into one of the two groups, and for which at least one post-treatment result was available. The “per-protocol population” consisted of 166 dogs, since eight dogs had major protocol deviations due to administration of forbidden concomitant therapies. The maximum number of dogs assessed by any one investigator was 23 (13% of the total).

There were no significant differences between groups in the pre-surgery variables or surgery data except for duration of surgery which was significantly longer (*P* = 0.037) in the robenacoxib group (Table 3). It was concluded that the two groups were adequately balanced at baseline.

**Table 3:** Demographic and surgery variables. Data are mean (SD) or number of dogs (%)

**Variable**	**Robenacoxib**	**Meloxicam**	**Total**	P value*
Number of dogs	118	56	174	
Age (years)	5.9 (3.9)	5.2 (4.1)	5.7 (3.9)	0.20
Body weight (kg)	20.8 (12.3)	19.0 (11.1)	20.2 (11.9)	0.42
Sex and neutered status				
Male not neutered	14 (11.9%)	8 (14.3%)	22 (12.6%)	0.37
Female not neutered	87 (73.7%)	40 (71.4%)	127 (73.0%)	
Male neutered	5 (4.2%)	0 (0%)	5 (2.9%)	
Female neutered	12 (10.2%)	8 (14.3%)	20 (11.5%)	
Pre-surgery pain (VAS)				
▪ At rest	2.0 (5.4)	1.7 (2.3)	1.9 (4.6)	0.21
▪ At palpation/manipulation	5.2 (10.2)	4.2 (6.3)	4.9 (9.2)	0.64
Type of surgery**				
▪ Ovariectomy	37 (31%)	22 (39%)	59 (34%)	0.31
▪ Ovario-hysterectomy	28 (24%)	10 (18%)	38 (22%)	0.44
▪ Mammary chain excision	30 (25%)	15 (27%)	45 (26%)	0.85
▪ Gastro-intestinal surgery	5 (4%)	0 (0%)	5 (3%)	0.18
▪ Genito-urinary surgery	5 (4%)	2 (4%)	7 (4%)	1.0
▪ Thoracic surgery	1 (1%)	0 (0%)	1 (1%)	1.0
▪ Other soft tissue surgery	22 (19%)	10 (18%)	32 (18%)	1.0
Duration of surgery (hours)	0.85 (0.52)	0.68 (0.35)	0.79 (0.48)	0.037
Duration of intubation (hours)	1.4 (0.77)	1.2 (0.60)	1.3 (0.73)	0.20
Time between injection of robenacoxib or meloxicam and extubation (hours)	1.6 (0.81)	1.4 (0.63)	1.5 (0.76)	0.18
Duration of oral dosing (days)	11.3 (22.2)	10.9 (3.0)	-	0.92

Data are mean (SD) or number of dogs (%).

* Significance of differences between treatment groups (Kruskal-Wallis or Mann–Whitney U tests).

** Some dogs underwent two surgeries during the same anesthesia.

Case allocation was stratified according to investigator and anticipated duration of surgery. Numbers of dogs in the robenacoxib and meloxicam groups were respectively 87 and 44 for predicted duration < 1 hour, and 31 and 12 for > 1 hour. Differences were not significant (*P* = 0.57).

### Test treatments

All dogs received a single subcutaneous injection of robenacoxib or meloxicam prior to surgery. The targeted subcutaneous dosage of robenacoxib was 2.0 mg/kg. Actual mean (range) dosages received were 2.00 (0.40 - 2.40) mg/kg. The targeted subcutaneous dosage of meloxicam was 0.20 mg/kg, whilst actual mean (range) dosages were 0.21 (0.15 - 0.50) mg/kg.

After surgery, dogs received follow-up treatment with oral robenacoxib or meloxicam.

The targeted oral dosage of robenacoxib was 1–2 mg/kg administered once daily for 10–14 days. Mean (range) administered dosages were 1.39 (0.86 -2.16) mg/kg for a median (range) of 12 (0–15) days. The targeted oral dosage of meloxicam was 0.1 mg/kg. Mean (range) dosages administered were 0.10 (0.09 - 0.11) mg/kg for 12 (0–14 days). A total of 10 (8%) dogs in the robenacoxib group and 6 (11%) in the meloxicam group were dosed orally for shorter than the minimum 10 days defined in the protocol, due to owner mistake or withdrawal of the dog from the study.

### Rescue therapy and concomitant treatments

Although clinicians were authorized to provide rescue therapy at any time after surgery if needed, no dog received such therapy. Concomitant therapies included anesthesia drugs permitted in the protocol. Premedicants (number of cases) included: no drug (42), acepromazine (75), diazepam (41), diazepam and atropine (7), diazepam and glycopyrollate (4), acepromazine and glycopyrollate (3), acepromazine and atropine (1), and atropine (1). Anesthesia was induced with either thiopentone (123) or propofol (51), and maintained with either halothane (92) or isoflurane (82). There were no significant differences between groups.

Additional allowed concomitant treatments were primarily fluids (131) and injectable (103) or oral (104) antibiotics, with no differences between groups. The principal antibiotics were amoxicillin and cephalexin.

Eight dogs (five in the robenacoxib group, three in the meloxicam group) were administered a total of eight disallowed drugs, as follows: doxycycline (1), spiramycin + metronidazole (3), spiramycin + metronidazole + prednisolone (1), ketamine (1), carprofen (1) and dexamethasone (1). With the exception of cases receiving spiramycin + metronidazole, all were administered as a single dose.

### Efficacy endpoints

**Figure 1:**
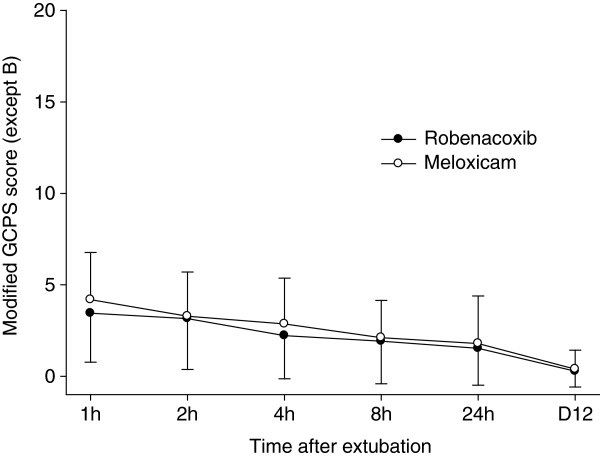
**Modified Glasgow Composite Pain Scale (GCPS) score (without part B) assessed by clinicians at defined times after extubation.** The score is a numerical rating scale ranging from 0 (minimum) to 20 (maximum). Data are mean and SD.

**Figure 2:**
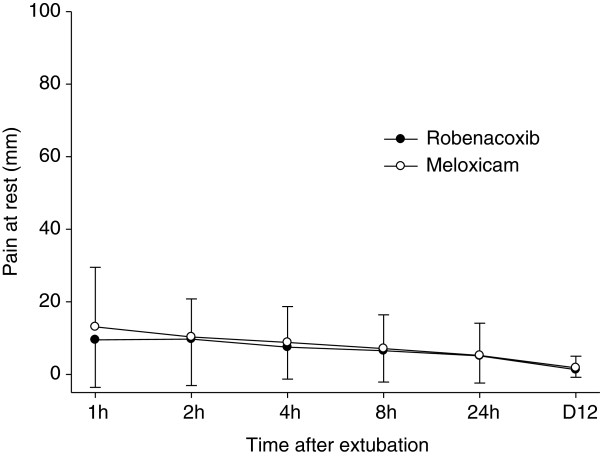
**Pain at rest at defined times after extubation.** Pain was assessed by clinicians using a 0–100 mm visual analogue scale. Data are mean and SD.

**Figure 3:**
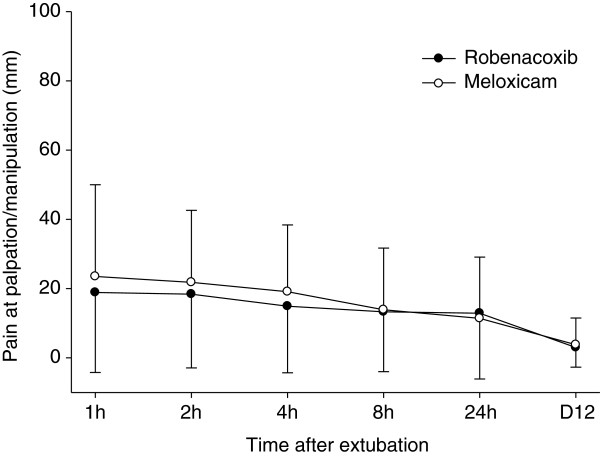
**Pain at palpation or mobilization at defined times after extubation.** Pain was assessed by clinicians using a 0–100 mm visual analogue scale. Data are mean and SD.

**Figure 4:**
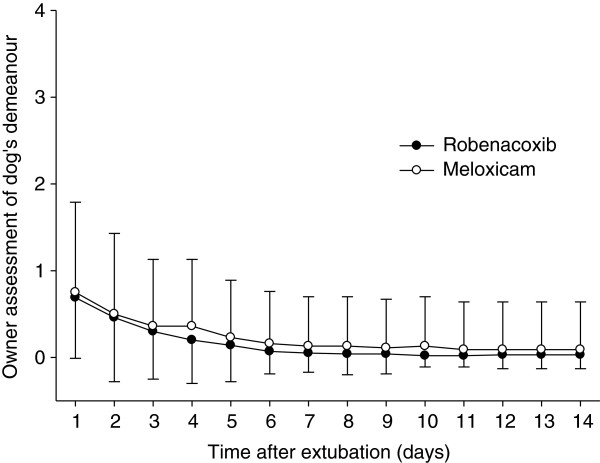
**The dog’s demeanor on days 1 to 14 after surgery.** The demeanor was assessed by owners using a 0–4 numerical rating scale. Data are mean and SD.

**Figure 5:**
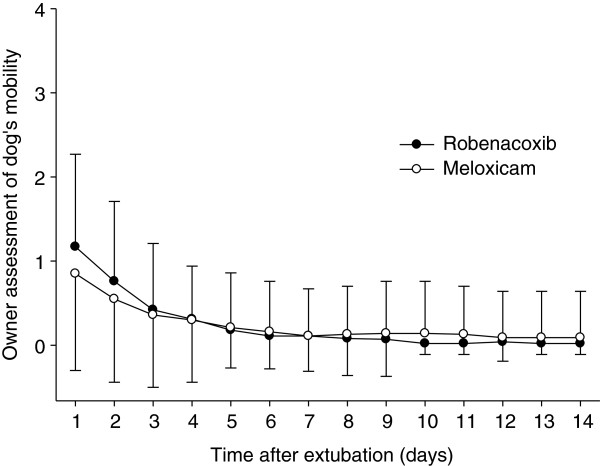
**The dog’s mobility on days 1 to 14 after surgery.** The mobility was assessed by owners using a 0–4 numerical rating scale. Data are mean and SD.

The primary endpoint was the “modified” GCPS score without part B (n = 153 dogs) since the mobility score (B) could not be assessed in 21 dogs. However similar results were obtained for the GCPS score with and without part B (Tables 4 and 5). Pain was well controlled in most cases in the post-operative period as indicated by mean modified GCPS scores less than 5.0 for dogs of both groups (maximum score = 20, Figure 1). Pain was most apparent on palpation/manipulation of the affected area rather than at rest (Figures 2 and 3). Scores were maximal at 1 hour and thereafter decreased slowly. For the owner assessments of demeanor and mobility, the highest mean scores were recorded on day 1 and decreased progressively thereafter (Figures 4 and 5).

**Table 4:** Efficacy endpoints

**Variable**	**Group**	**Estimate**	**Standard error**	**95% Confidence limits**		**P value ANOVA/RMANOVA**	**P value Mann–Whitney U**
				**Lower**	**Upper**		
**Primary endpoint**							
**Modified GCPS score (without B)**	Robenacoxib	1.65	0.12	1.42	1.90	0.12	0.29
	Meloxicam	1.97	0.18	1.63	2.35		
**Unmodified GCPS score (with B)**	Robenacoxib	2.35	0.17	2.03	2.71	0.13	0.72
	Meloxicam	2.79	0.26	2.31	3.34		
**Secondary endpoints**							
**Pain at rest**	Robenacoxib	2.62	0.31	2.06	3.28	0.20	0.54
	Meloxicam	3.30	0.49	2.43	4.39		
**Pain at palpation/manipulation**	Robenacoxib	4.34	0.53	3.39	5.50	0.28	0.71
	Meloxicam	5.29	0.84	3.82	7.19		
**Global efficacy score**	Robenacoxib	0.45	0.06	0.33	0.58	0.28	0.22
	Meloxicam	0.56	0.09	0.39	0.76		
**Global inflammation score**	Robenacoxib	1.11	0.25	0.66	1.67	0.12	0.59
	Meloxicam	1.76	0.42	1.04	2.74		
**Owner assessment: demeanor**	Robenacoxib	0.10	0.02	0.07	0.13	0.96	0.62
	Meloxicam	0.10	0.02	0.06	0.14		
**Owner assessment: mobility**	Robenacoxib	0.10	0.03	0.05	0.16	0.17	0.098
	Meloxicam	0.05	0.03	-0.01	0.12		

GCPS = Glasgow Composite Pain Scale.

Data are ANOVA or RMANOVA estimates for group means, standard errors and confidence intervals. P values for group comparisons are from ANOVA or RMANOVA and Mann–Whitney U tests.

**Table 5:** Efficacy endpoints: non-inferiority analysis, ANOVA or RMANOVA estimates for group quotients robenacoxib/meloxicam

**Variable**	**Estimate (quotient)**	**Standard error**	**95% Confidence limits**		**P value ANOVA/RMANOVA**	**P value for normality (Shapiro-Wilk)**
			**Lower**	**Upper**		
**Primary endpoint**						
**1/Modified GCPS score (without B)**	1.12	0.08	0.97*	1.29	0.12	<.0001**
**1/Unmodified GCPS score (with B)**	1.13	0.09	0.96*	1.32	0.13	0.0005**
**Secondary endpoints**						
**1/Pain at rest**	1.19	0.16	0.91*	1.55	0.20	<.0001**
**1/Pain at palpation/manipulation**	1.18	0.18	0.87*	1.58	0.28	0.0049**
**1/Global efficacy score**	1.08	0.07	0.94*	1.23	0.28	<.0001**
**1/Global inflammation score**	1.31	0.23	0.93*	1.85	0.12	0.058
**1/Owner assessment: demeanor**	1.00	0.02	0.95*	1.05	0.96	0.010**
**1/Owner assessment: mobility**	0.95	0.03	0.89*	1.02	0.17	0.010**

GCPS = Glasgow Composite Pain Scale.

All data were log transformed for the ANOVA/RMANOVA analyses.

Values are reciprocals of scores, thus values >1.00 indicate lower scores (higher efficacy) for robenacoxib versus meloxicam.

* Non-inferiority of robenacoxib to meloxicam shown (lower confidence limit >0.80).

** Data were not normally distributed after log transformation.

Overall group means and group comparison P values using the non-parametric Mann–Whitney *U* test are presented for all endpoints in Table 4. For both the primary and all six secondary efficacy endpoints, there were no significant differences between the robenacoxib and meloxicam groups.

In the non-inferiority analysis (Table 5), non-inferiority of robenacoxib relative to meloxicam was present, as indicated by lower confidence interval values >0.80, for the primary endpoint and all six secondary endpoints. However, in spite of log transformation of the data, the models deviated significantly from a normal distribution for all endpoints except for inflammation. For the primary endpoint, the modified GCPS score (without part B), the relative efficacy of robenacoxib/meloxicam was 1.12 with a 95% confidence interval of 0.97-1.29. In the RMANOVA model, for the modified GCPS score, significant effects of the following covariates persisted in the final model: time (*P* < 0.0001); country (*P* < 0.007) and body weight (*P* = 0.020).

Similar results were obtained using the “per-protocol” population, with non-inferiority proven for the primary endpoint and five of the six secondary endpoints (data not shown). For the primary endpoint (modified GCPS score) using the “per-protocol population”, the relative efficacy of robenacoxib/meloxicam was 1.08 with a 95% confidence interval of 0.91-1.28.

In some other studies, rescue therapy was administered automatically if a minimum GCPS score was reached, for example ≥5 for the modified score (without part B) or ≥6 for the unmodified score (with part B) [15,16]. Therefore, although not planned in the protocol, we evaluated the number of dogs in each group which had defined scores. P values were calculated with the Fisher Exact test. The number (percentage of total) dogs with a modified GCPS score (without B) ≥5 was 47 (39.8%) for robenacoxib and 26 (46.4%) for meloxicam (*P* = 0.42); ≥6 was 29 (24.6%) for robenacoxib and 22 (39.3%) for meloxicam (*P* = 0.052); ≥7 was 24 (20.3%) for robenacoxib and 17 (30.4%) for meloxicam (*P* = 0.18); and ≥8 was 17 (14.4%) for robenacoxib and 12 (21.4%) for meloxicam (*P* = 0.28).

The number (percentage of total) dogs with an unmodified GCPS score (with B) ≥6 was 60 (50.8%) for robenacoxib and 35 (62.5%) for meloxicam (*P* = 0.19); ≥7 was 47 (39.8%) for robenacoxib and 29 (51.8%) for meloxicam (*P* = 0.15); and ≥8 was 40 (33.9%) for robenacoxib and 23 (41.1%) for meloxicam (*P* = 0.40).

### Plasma cortisol concentration

Plasma cortisol concentrations prior to dosing and up to 8 hours after extubation are presented in Table 6. Significant and quantitatively similar increases compared with pre-anesthesia values were obtained in both groups (*P* < 0.0001, Wilcoxon test). Log-transformed cortisol concentrations fulfilled normal distribution assumptions in the ANOVA (*P* = 0.30) but not RMANOVA (*P* = 0.0001) analysis. Using ANOVA, there were no significant difference between the robenacoxib and meloxicam groups (*P* = 0.20). Non-inferiority was demonstrated in the ANOVA analysis, the mean quotient (robenacoxib/meloxicam) of the reciprocal of cortisol concentrations was 0.92 (95% confidence interval, 0.84-1.02). There were significant model effects in the ANOVA for country (*P* = 0.027), baseline (*P* <0.0001), duration of intubation (*P* = 0.0021) and type of surgery (*P* = 0.0046).

**Table 6:** Plasma cortisol concentrations (ng/mL) prior to and at times following surgery

**Time (h)**	**Robenacoxib**		**Meloxicam**	
	**Mean**	**SD**	**Mean**	**SD**
**Pre-surgery**	69.8	41.62	76.1	52.19
**0**	136.6	53.90	125.9	51.13
**1**	159.8	74.76	140.4	60.87
**2**	145.7	76.30	132.9	74.47
**4**	101.6	60.00	98.3	47.23
**8**	88.3	46.78	85.1	44.03
**Mean (post-surgery)**	116.0	51.21	108.5	42.26

### Tolerability

A total of 17 of the 118 dogs (14%) receiving robenacoxib and 12 of the 56 dogs (21%) receiving meloxicam had reported adverse events (*P* = 0.26). Most adverse events were classified as benign or moderate. The most frequent reports concerned the gastrointestinal tract (vomiting, diarrhea, dark or soft stools). One death occurred in a 12 year old dog in the meloxicam group due to pancreatitis. This event was classified as unrelated to treatment. Five adverse events were classified as severe, of which two were judged to be unrelated to treatment and two classified as unknown relationship to treatment. Both of the latter were in the robenacoxib group: one comprised dermatitis in the surgical area and the second involved hematoma linked to the surgery. A fifth case, also in the robenacoxib group, presented with vomiting 15 minutes after extubation and was classified as possibly linked to treatment. This dog received metoclopramide and recovered completely without alteration to robenacoxib dosing.

Pain at the site of subcutaneous injection was minimal, being reported as absent in 109 (92%) and present in 9 (8%) of dogs receiving robenacoxib, and absent in 47 (85%) and present in 8 (15%) of dogs receiving meloxicam. Differences between groups were not significant (*P* = 0.17).

There were no changes in buccal mucosal bleeding time at T1 + 24 h compared to prior to surgery (T0) with either drug. Mean ± SD bleeding times at T0 and T1 + 24 h were respectively 131 ± 63 and 131 ± 56 min with robenacoxib (*P* = 0.80), and 143 ± 65 and 122 ± 59 min with meloxicam (*P* = 0.091). There was no significant difference between groups at baseline (*P* = 0.91) or at 24 hours (*P* = 0.20).

For clinical chemistry and hematology variables, there were isolated incidents of statistical significance at 24 hours which were judged not be relevant clinically: white cell counts higher with robenacoxib and plasma urea concentrations higher with meloxicam. There were no significant differences between groups for change from baseline analyses.

## Discussion

The main conclusion of this study is that a treatment regimen of a single subcutaneous injection of robenacoxib before surgery, followed by once daily administration of robenacoxib tablets for 12 days, was well tolerated and had statistically non-inferior efficacy in comparison with meloxicam for the management of pain and inflammation associated with soft tissue surgery in dogs. A variety of methods are available for the clinical assessment of pain in animals. In this study, the primary endpoint was the clinician’s assessment of the GCPS [13]. At the time of initiation of the study, weighing factors for the indices had not yet been published [1,17], and therefore unweighted results are reported. The results of the primary endpoint were supported by six secondary endpoints. No significant differences between groups were observed using the non-parametric Mann–Whitney *U* test for the primary and all six secondary efficacy variables. Non-inferior efficacy of robenacoxib compared to meloxicam was also demonstrated statistically for both the primary and all six secondary efficacy using ANOVA and RMANOVA analyses. However the reliably of those ANOVA and RMANOVA analyses is uncertain since the data deviated significantly from a normal distribution, in spite of log transformation, for all endpoints except inflammation. In addition the GCPS is the sum of ordinal or ranking scales and is not an interval scale, and therefore in principle non-parametric statistics should be used. However to our knowledge no suitable non-parametric methods exist for non-inferiority analysis, which was the primary objective of the study. Therefore a combination of non-parametric and parametric tests was used, and using both approaches provides greater confidence that robenacoxib had non-inferior efficacy compared to meloxicam.

The major limitations of the study are discussed below. First, although the dogs were assessed reasonably intensively by the clinician in the first 24 hours after surgery while hospitalized, the follow-up from day 2 onwards was sparse and consisted mainly of daily assessments of the dog’s demeanor and mobility by the owner. A final examination was made by the clinician when the stitches were removed at day 12. Second, although the clinicians were masked to treatment groups, via the use of a dispenser, the dog owners were not blinded. For this and other reasons, the primary endpoint of the study was based on the clinician and not the owner scores. Third, no specific assessment was made of sedation, and therefore we do not know if sedation could have confounded the efficacy assessments. However signs consistent with sedation were only reported in one dog (“apathy” in the robenacoxib group) and to our knowledge neither meloxicam nor robenacoxib have any reported sedative properties. No sedative effects were reported with administration of high dosages of robenacoxib (40 mg/kg daily for 1 month) to dogs [5]. Fourth, although the clinicians were authorized to provide rescue therapy at any time after surgery if needed, no dog received such therapy. Other authors have automatically administered rescue therapy to dogs if a minimum score was reached, for example a modified GCPS score (without part B) ≥5 or an unmodified score (with part B) ≥6 [15,16]. Although not planned in the protocol of this study, we determined that the number (percentage of total) of dogs with a modified GCPS score ≥5 was 47 (39.8%) in the robenacoxib group and 26 (46.4%) in the meloxicam group (*P* = 0.42). For the unmodified score (with part B), the number of dogs with a score ≥6 was 60 (50.8%) in the robenacoxib group and 35 (62.5%) in the meloxicam group (*P* = 0.19). Therefore different investigators may have concluded that many of the dogs in the study required additional analgesia. The relatively high number of dogs with GCPS scores above the thresholds defined above is probably due to the fact that opiates were not used in any dog. Optimal control of post-operative pain requires mixed therapy with NSAIDs and opioids [2]. Fifth and last, the limitations of non-inferiority studies using positive controls are well-known [18]. In this case, however, use of a placebo would have raised ethical and recruitment issues in this study conducted in France and Germany since a number of NSAIDs are registered for peri-operative use in dogs in the EU and are widely used. Meloxicam was selected as the positive control since it is registered and extensively used in dogs, and its efficacy administered by injection prior to surgery in dogs undergoing soft tissue surgery has been proven via superiority to butorphanol or placebo [9-11]. To our knowledge there exist no published data on the efficacy of oral meloxicam to treat post-operative pain for 12 days in dogs, as used in our study. The efficacy of meloxicam versus placebo was demonstrated for up to 72 hours after ovariohysterectomy [11]. However, oral 0.1 mg/kg meloxicam had significant efficacy in the urate synovitis model in dogs [19]. Therefore we conclude that there is satisfactory evidence for the efficacy of meloxicam to control pain and inflammation in dogs undergoing soft tissue surgery, and thus it was a suitable positive control for this study. However in none of the above mentioned published studies was the GCPS used, as in our study. In optimally designed non-inferiority studies, the methods and outcome measures should be similar to those used in the original studies of the active control [18]. Superiority of deracoxib versus placebo, and firocoxib versus a negative control, was reported recently from two studies in dogs undergoing soft tissue using similar methods to our study including use of the GCPS [20,21]. The frequency of rescue therapy in those studies with treatment as a single dose pre-surgery followed by 2 days post-surgery was higher [deracoxib 2/16 (12.5%), placebo 9/16 (56.25%), firocoxib (16.4%), negative control (50.6%)] than in our study with robenacoxib (0%) or meloxicam (0%).

A feature of our study was the choice of a non-inferiority threshold (δ) value of 0.20. The δ value should reflect the largest margin that is clinically acceptable. In fact the results show that non-inferior efficacy of robenacoxib to the positive control would also have been achieved if we had defined δ = 0.05 for the primary endpoint i.e. with a maximum of 5% difference in modified GCPS scores. Furthermore, robenacoxib had numerical superiority to meloxicam for the primary endpoint (relative efficacy 1.12) and for five of the secondary endpoints (range 0.95 to 1.31). Therefore, the data support the conclusion of non-inferior efficacy.

In both groups plasma cortisol concentrations were approximately doubled after surgery, relative to pre-anesthesia values, and had not returned to control values at the last time point of 8 hours after extubation. The extent and duration of the observed rise in cortisol concentrations are consistent with published literature in which increases by factors of 2 to 3 lasting 2 to 4 hours are reported [22-24]. There were no significant differences between the treatment groups in cortisol concentrations. In a previous study, no change in plasma cortisol concentrations was noted with meloxicam in dogs undergoing mammary gland excision [25].

Robenacoxib and meloxicam were both well tolerated in this study. Although adverse effects were reported in 29 of 174 dogs, most were assessed as mild or benign and classified as either not or only possibly treatment related. The adverse effects reported most frequently were occasional vomiting and loose stools. There were also no biologically relevant changes in hematology and biochemistry variables, and no significant change in buccal mucosa bleeding time, with either robenacoxib or meloxicam. The latter result is not surprising for robenacoxib, since it does not inhibit COX-1 at recommended dosages in dogs [4,6] or affect buccal bleeding time at therapeutic or elevated dosages in healthy dogs [5]. Buccal bleeding time also did not increase with meloxicam, although this might be expected from the fact that 0.3 mg/kg meloxicam by injection significantly inhibits COX-1 in dogs [4]. No significant differences in buccal mucosa bleeding time were noted previously in dogs receiving meloxicam compared to placebo in dogs undergoing abdominal surgery [9,10,26].

The rationale for the development of highly COX-2 selective NSAIDs such as robenacoxib is that they should offer the same efficacy but better safety than older less selective NSAIDs such as meloxicam [7]. The finding of no significant differences in tolerability between robenacoxib and meloxicam in this study is not surprising, however, since the study was underpowered to detect differences in safety parameters, with only 174 dogs and a relatively short treatment duration (maximum 16 days). In addition we did not include specific safety investigations, for example gastroscopy, which might have revealed differences in tolerability as noted previously for other NSAIDs [27].

## Conclusions

In this clinical trial, a treatment regimen of robenacoxib, consisting of a single subcutaneous injection prior to surgery (2 mg/kg) followed by once daily administration of tablets post-operatively at 1–2 mg/kg for 12 days, had good tolerability and non-inferior efficacy compared to meloxicam for the control of pain and inflammation in dogs undergoing soft tissue surgery.

### Availability of supporting data

Due to the confidentiality of records from owners and veterinarians in this clinical trial, the data base cannot at present be made freely available. Please contact the authors if you require access to selected parts of the data.

## Abbreviations

ANOVA: Analysis of variance; COX: Cyclo-oxygenase; D: Day; EU: European Union; GCPS: Glasgow Composite Pain Scale; h: Hour; LOCF: Last observation carried forward; NSAID: Non-steroidal anti-inflammatory drug; RMANOVA: Repeated measures analysis of variance; T: Time; VAS: Visual analogue scale.

## Competing interests

All authors are employees of Novartis Animal Health Inc., and stock holders of Novartis Inc., which manufactures and distributes robenacoxib (Onsior®). The authors declare that they have no further competing interests.

## Authors’ contributions

PG and JNK conceived and designed the study. PG managed the study (clinical trial). WS analyzed the data. PG and JNK interpreted the data. JNK wrote the paper. All authors read and approved the final manuscript.
